# Identification of LAG3 high affinity aptamers by HT-SELEX and Conserved Motif Accumulation (CMA)

**DOI:** 10.1371/journal.pone.0185169

**Published:** 2017-09-21

**Authors:** Mario Martínez Soldevilla, Sandra Hervas, Helena Villanueva, Teresa Lozano, Obdulia Rabal, Julen Oyarzabal, Juan José Lasarte, Maurizio Bendandi, Susana Inoges, Ascensión López-Díaz de Cerio, Fernando Pastor

**Affiliations:** 1 Aptamer Platform, Molecular Therapeutics Program, Center for Applied Medical Research, (CIMA), Pamplona, Spain; 2 Instituto de Investigación Sanitaria de Navarra (IDISNA), Recinto de Complejo Hospitalario de Navarra, Pamplona, Spain; 3 Program Immunology and Immunotherapy, Center for Applied Medical Research, (CIMA), University of Navarra, Pamplona, Spain; 4 Small Molecule Discovery Platform, Molecular Therapeutics Program, Center for Applied Medical Research (CIMA), Pamplona, Spain; 5 Section on Hematology/Oncology, Department of Internal Medicine, Comprehensive Cancer Center, Wake Forest University Baptist Healthcare Center, Winston-Salem, NC, United States of America; 6 Section of Hematology/Oncology, Department of Internal Medicine, W.G Hefner VA Medical Center, Salisbury/Charlotte, NC, United States of America; 7 Department of Immunology and Immunotherapy, University Clinic of Navarra, Pamplona, Spain; Duke University, UNITED STATES

## Abstract

LAG3 receptor belongs to a family of immune-checkpoints expressed in T lymphocytes and other cells of the immune system. It plays an important role as a rheostat of the immune response. Focus on this receptor as a potential therapeutic target in cancer immunotherapy has been underscored after the success of other immune-checkpoint blockade strategies in clinical trials. LAG3 showcases the interest in the field of autoimmunity as several studies show that LAG3-targeting antibodies can also be used for the treatment of autoimmune diseases. In this work we describe the identification of a high-affinity LAG3 aptamer by High Throughput Sequencing SELEX in combination with a study of potential conserved binding modes according to sequence conservation by using 2D-structure prediction and 3D-RNA modeling using Rosetta. The aptamer with the highest accumulation of these conserved sequence motifs displays the highest affinity to LAG3 recombinant soluble proteins and binds to LAG3-expressing lymphocytes. The aptamer described herein has the potential to be used as a therapeutic agent, as it enhances the threshold of T-cell activation. Nonetheless, in future applications, it could also be engineered for treatment of autoimmune diseases by target depletion of LAG3-effector T lymphocytes.

## Introduction

LAG3 (CD223) was discovered in the 90s as a CD4 homologous receptor. Both LAG3 and CD4 bind to MHC-II, even if through different structure motifs [1]. The immunomodulatory function of LAG3 receptor was deciphered years later with the generation of LAG3-knockout mice [2]. As these mice age, they show changes in the immune cell compartment resulting in the increase of multiple cell types including T lymphocytes, B cells, macrophages, granulocytes, and dendritic cells [3]. LAG3-/- NOD mice are also more prone to develop diabetes [4]. In vitro LAG3-/- lymphocytes show a lower threshold of activation, although they exhibit increased cell death [5]. Taken together, this data indicates LAG3 acts as an immune-checkpoint receptor quenching the immune response, becoming a very attractive “druggable” receptor for cancer immunotherapy, especially in combination with PD1 and/or CTLA4 blockade. There are currently several ongoing clinical trials with LAG3-targeted immunotherapy [6]. The earliest clinical studies with LAG3 were done (some of them are still ongoing) with IMP321, which is a recombinant protein with the LAG3 extracellular domain attached to the Fc portion of IgG1 (LAG3-Ig) [7]. Pre-clinical studies with LAG3-Ig have shown that the mechanism acts mainly by favoring matured dendritic cells to favor Th1 responses; it has been used as adjuvant of cancer vaccines [8]. More recently, LAG3 receptor-blocking monoclonal antibodies have been developed showing an enhancement of tumor immunity in combination with PD1 blockade [9]. Human LAG3 antibodies are being used in clinical trials for different types of tumors in combination with other immune-stimulatory treatments (clinicaltrials.gov).

Paradoxically, LAG3 receptor is also a desirable target for autoimmune diseases and allograph rejection treatments. It has recently been shown that mice that lacked LAG3 expression on Tregs exhibited reduced autoimmune diabetes [10], indicating an opposite role of LAG3 in Treg. Besides, a clinical trial with anti-LAG3 monoclonal antibody for the treatment of psoriasis has been initiated. This anti-LAG3 antibody has been optimized to induce selective depletion of cells expressing LAG3 by antibody-dependent cell cytotoxicity. This approach triggers the depletion of reactive T lymphocytes in autoimmunity and allograph transplants [11].

All the available LAG3-targeting therapeutic agents described so far and those used in the clinic are proteins which are cell-based derived products. Protein-derivate reagents have some limitations and are highly expensive to produce in GMP grade, increasing the prices of this type of therapeutics [12]. In this work we describe the first anti-LAG3 aptamer. Aptamers are single-strand oligonucleotides selected through a combinatorial process named Systematic Evolution of Ligands by Exponential Enrichment (SELEX) [13–14]. The selected aptamers can be chemically synthesized. In the last few years aptamers are getting into the therapeutic arena [15]. The use of aptamers as new therapeutic platforms in cancer immunotherapy is even more recent. Herein we combine High Throughput Sequencing (HTS) SELEX [16] with sequence-conserved motif along with in silico predicted 3D structure to identify a high affinity aptamer against murine LAG3 receptor. We describe a novel and straightforward strategy based on the Conserved Motif Accumulation (CMA) that could be used for the selection of further aptamers against other targets. Our results suggest that aptamer species with a higher accumulation of potential binding motifs are likely to have a higher probability of being better binders.

## Results

### Identification of LAG3 aptamers by HT-SELEX

**Fig 1:**
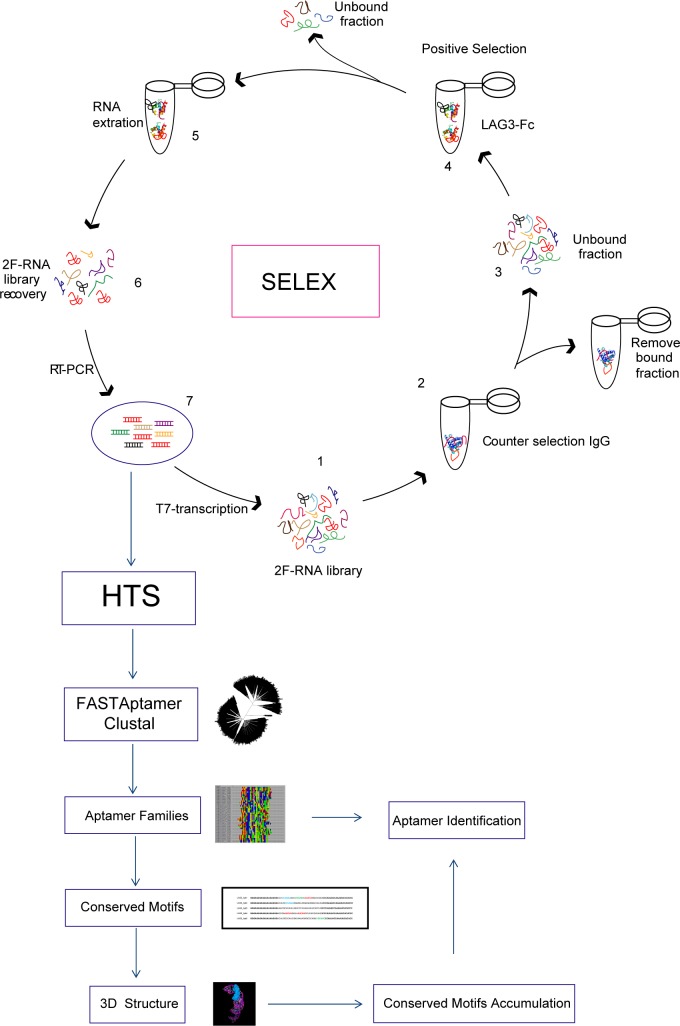
Proposed workflow to identify high affinity aptamers using HT-SELEX combined with Conserved Motif Accumulation (CMA).

The workflow used for LAG3 aptamer selection is shown in Fig 1. Seven rounds of selection were performed against the LAG3-Fc recombinant protein by SELEX. The initial DNA library was transcribed in vitro in RNA with 2’-fluoro-pyrimidines to increase the stability of the aptamers in light of future in vivo therapeutic applications. The 31-nucleotide DNA library has two constant flanking regions at the 5’ end (GGGGAATTCTAATACGACTCACTATAGGGAGAGAGATATAAGGG) and at the 3’ end (CCCATTACCAAATTCTCTCCC) to facilitate the transcription to 2F’-RNA with a T7 mut-polymerase and the amplification by polymerase chain reaction (PCR) of the enriched aptamer species in each round of selection.

**Fig 2:**
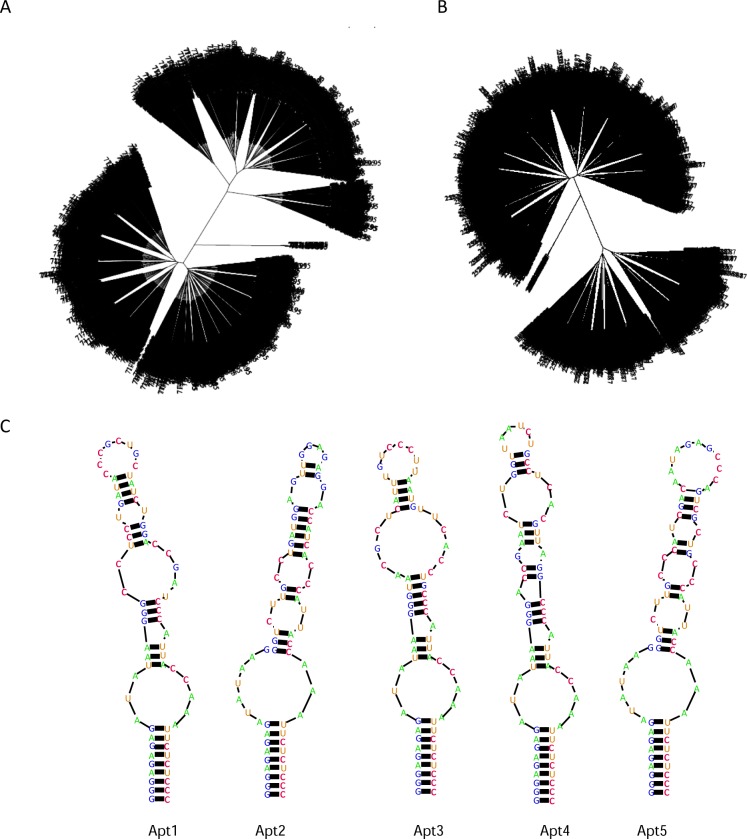
HT-SELEX Aptamer selection analysis. A) Phylogenetic representation of every main aptamer species obtained from deep sequencing by Ion Torrent at rounds R06 and R07 after multiple alignment using Clustal W and FASTAptamer. B) Secondary structure predicted by RNAstructure of the five most populated aptamers.

The whole selection process was done against the murine recombinant protein LAG3-Fc immobilized in sepharose beads. Bound aptamer species were pulled down by precipitation with the sepharose beads. Counter-selection was performed in each round with IgG protein to avoid the enrichment of aptamers that could bind to the Fc region of the immunoglobulin. Selection conditions and restriction were increased along the selection process as indicated in S1 Table, diluting the concentration of targeted recombinant protein and RNA library as well as increasing the washing time of protein-bound sepharose beads along the selection process. Recovery of aptamer selection was considerably reduced in round seven, so we decided to stop the selection in this round. Aptamer library from rounds six and seven was deep sequenced via Ion Torrent. Five major aptamer species were highly enriched in round 7; the fold change of these aptamers from round 6 to round 7 is shown in S1 Fig. The alignment family clusterization from round 6 and round 7 aptamer library were analyzed using the FASTAptamer software [17] and ClustalW [18]. The whole FASTAptamer analysis of rounds 6 and 7 as well as the grade of enrichment of each aptamer species is indicated in S2 and S3 Tables. The phylogenetic relation of each aptamer was also determined (Fig 2A). The predicted secondary structure of each of the five identified aptamers highly enriched in the HT-SELEX was obtained by using the RNAstructure software (Fig 2B).

### Prioritized LAG3 aptamers by Conserved Motif Accumulation (CMA)

**Fig 3:**
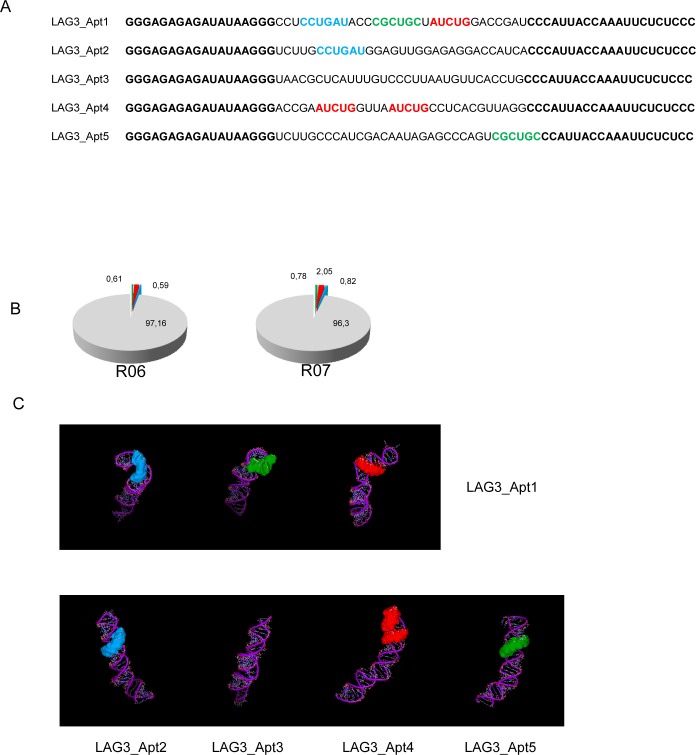
Analysis of conserved sequence motifs in LAG3 aptamers. A) Presence of three sequence motifs–CGCTGC (green), ATCTG (red) and CCTGAT (blue)–identified in the most abundant LAG3 aptamer species. B) Frequencies of LAG3 sequence motifs within the whole aptamer library at rounds 6 and 7. C) Location of LAG3 conserved sequence motifs in the predicted 3D structure of the five most abundant aptamers.

Analyzing the sequence of the five most abundant aptamers obtained from the HTS analysis of rounds 6 and 7, we identified three major motifs (CCTGAT, CGCTGC, ATCTG) that were quite conserved in all the aptamers but Apt3 (Fig 3A). Apt1 is the most populated one and has all three motifs in the 31-nucleotide variable region. The frequency of these motif sequences was also analyzed for all aptamers obtained at rounds 6 and 7 from HTS analysis. As shown in Fig 3B ATCTG is the most abundant motif among all considered, present in 2.05% of all sequences in round 7 and 1.62% of round 6.

The fact that Apt1 contains all motif sequences indicates that this aptamer could interact with LAG3 protein at different regions, probably recognizing three different aptatopes simultaneously, similarly to the complementary determined regions (CDR) of antibodies. In order to assess the impact of the presence and positioning of these structural motifs within the aptamer, we decided to model the 3D aptamer structure using Rosetta software Fragment Assembly of RNA with Full-Atom Refinement (FARFAR) [19]. Starting from the predicted 2D structure (Fig 2B), a total of 250 low resolution 3D models were required. Then, those models having the top lowest energy scores (energy score < -350) were clustered based on their structure similarity. The most populated cluster was chosen as a representative set of 3D models, and from there we selected the one with the lowest energy score that would likely reflect the native RNA structure. Conserved sequence motifs were visually inspected to determine similar structural motifs and relative orientation (Fig 3C).

### Identified LAG3 aptamers bind to rmLAG3-Fc protein with high affinity

**Fig 4:**
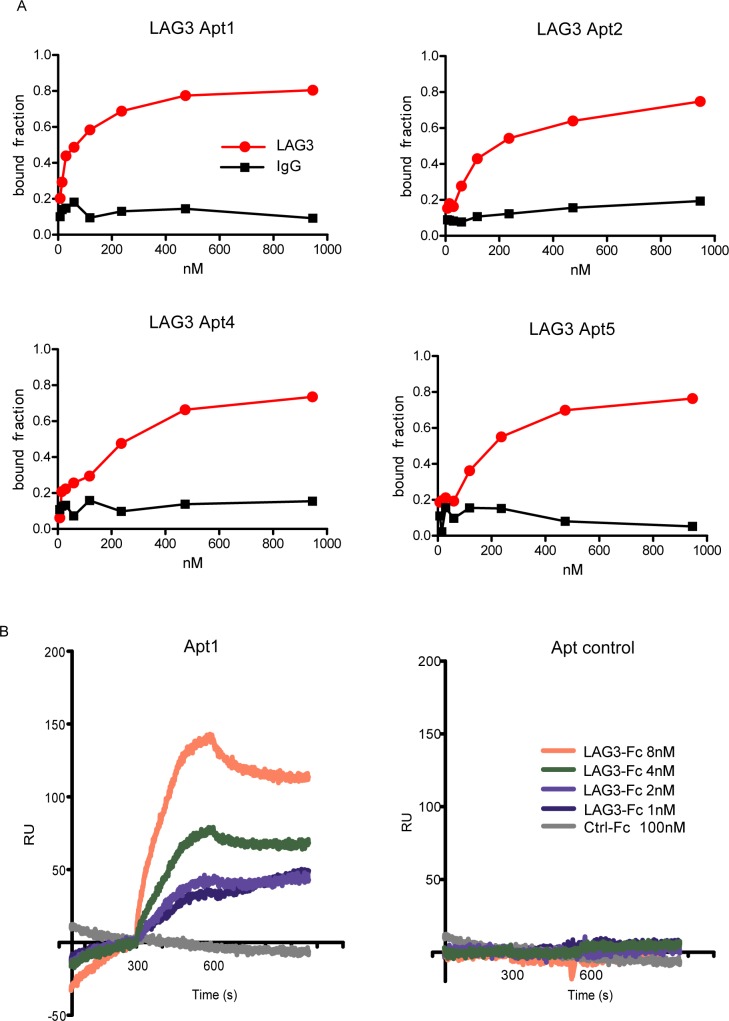
Binding affinity and specificity of LAG3 aptamers. A) Nitrocellulose filter-binding assay of LAG3 Apt 1, 2, 4 and 5 to the LAG3 recombinant protein. B) Plasmon Resonance (SPR) of LAG3 Apt1 at different concentrations of LAG3 recombinant protein.

Based on their predicted 3D structure and degree of motif conservation, four aptamers were selected for binding affinity characterization (Apt1, Apt2, Apt4, Apt5). Affinity of each aptamer was determined by filter binding assay with P^32^ labeled aptamers. The R0 library was used as a control on the experiment. Non-binding to IgG protein was detected confirming that the aptatopes of the four selected aptamers are within the LAG3 motif of the recombinant protein (Fig 4A). The aptamer affinities determined by filter binding and by apparent Kd ranking order were: Apt1 Kd 20 nM > Apt2 Kd 50 nM > Apt4 Kd 100 nM > Apt5 Kd 200 nM. Apt1, the one that contains the three conserved motifs, is the aptamer showing the highest affinity for LAG3-Fc recombinant protein. This observation could indicate that the binding interaction of Apt1 is a cooperative process where different aptatopes of the LAG3-Fc targeted protein are recognized by the same aptamer. Disappointingly, the crystal structure of LAG3 is not currently available. Therefore, no docking experiments on the binding of these aptamers could be carried out. The affinity for LAG3-Fc protein was also confirmed by Surface Plasmon Resonance (SPR) at different concentrations of LAG3-Fc recombinant protein (8, 4, 2 and 1 nM). A recombinant protein with the same Fc tag was also run in the SPR chip to ensure that the binding was against the LAG3 domain. As it can be depicted in Fig 4B no binding to control Fc protein is detected by SPR. Apt1 was able to detect concentration of LAG3-Fc protein as low as 1 nM by SPR. R0 library was also used as an aptamer control, showing no binding to LAG3-Fc protein (Fig 4B). From the SPR analysis Kon = 1x10^3^ Ms^-1^ and Koff< 2.1x10^-4^ s^-1^have been extracted. The apparent Kd obtained from the SPR results is 2.7 nM, lower than the one detected P^32^ binding assay. The disparities in the Kds might be due to the intrinsic conditions of each assay. For instance, in SPR the aptamer is immobilized to the SPR chip by biotin, while in the P^32^ binding assay both substrates (aptamer and ligand) are free in solution.

### LAG3 RNA aptamers recognize mouse LAG3 on the cell surface of T lymphocytes

**Fig 5:**
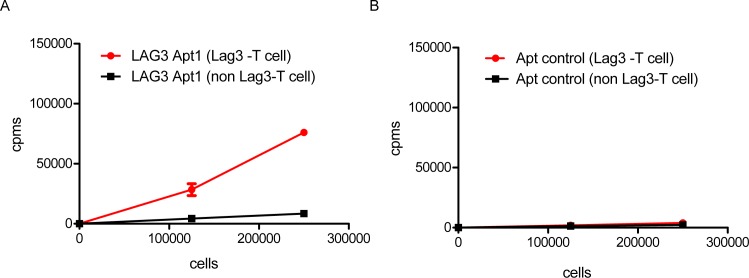
LAG3 Apt1 binds to LAG3-expressing cells. LAG3-Apt1 dimer labeled with P^32^ as described in methods to different amount of T lymphocytes expressing LAG3 (activated) in comparison with non-expressing LAG3 T lymphocytes (non-activated). n = 3. See also S3 Fig.

We decided to focus on Apt1 for further characterization. Not all the aptamers that bind to the soluble recombinant protein might bind to the native protein; changes in glycosylation pattern or in the folding structure associated with the addition of the Fc tag could preclude the recognition of the aptamer to the native protein. Therefore, it is important to ensure that the aptamer that binds to recombinant protein LAG3-Fc will also recognize the native LAG3 receptor expressed on T lymphocytes. Since LAG3 antibodies that are currently used in the clinic are bivalent molecules, we decided to dimerize LAG3 Apt1. To that end, each LAG3 Apt1 monomer was extended by in vitro transcription with 21 complementary sequences at the 3’ end. Dimerization was done by hybridization of the 21 complementary sequences (S2 Fig). In order to test the binding of LAG3 Apt1 dimer to LAG3-expressing lymphocytes, we radiolabeled the aptamer with P^32^; aptamer was in vitro transcribed, spiked with α-ATP (P^32^), and purified by PAGE. An equivalent control aptamer with a randomized variable region sequence P^32^-labeled was also used as control. We decided to use a radiolabeled aptamer to ensure that the aptamer structure was not affected by the addition of extra cargos such as fluorochromes, which in some cases could hamper its affinity. LAG3 is induced on activated T lymphocytes, while naïve T cells barely express LAG3, which was verified by flow cytometry analysis (S3 Fig). Thus, we used activated CD8 T lymphocytes OT-I activated with SIINFEKL and non-activated CD8 OT-I lymphocytes as control. To confirm that the binding was dependent on LAG3 expression, we performed serial dilutions of the cells. As we can observe in Fig 5, radiolabeled Apt1 binds only to LAG3-expressing lymphocytes (activated T cells), while the control aptamer does not bind at all. The measured counts per million (cpm) associated with the binding of Apt1 to the T lymphocytes decreases proportionally to the dilution of cells.

### LAG3 RNA aptamer enhance CD4 and CD8 lymphocyte activation in vitro

**Fig 6:**
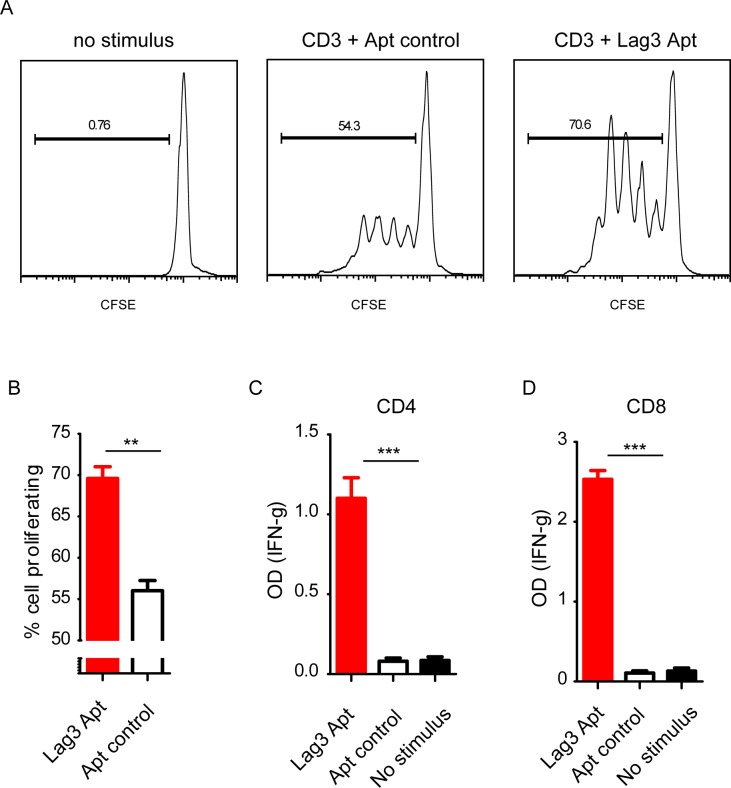
LAG3 Apt1 enhances T-cell activation in vitro. A) CD4 T-cell proliferation determined by CFSE dilution. Isolated CD4 lymphocytes labeled with CFSE and stimulated with anti-CD3 antibody and cultured with 10:1 ratio of splenocytes as feeders. B) Proliferation rate of CD4 lymphocytes as in A, n = 3. C) IFN-γ determined by ELISA in the supernatant of CD4 lymphocytes cultured as in A. n = 3 D) IFN-γ determined by ELISA in the supernatant of CD8 lymphocytes cultured as in A. n = 3.

LAG3 has been underscored as an important immune-checkpoint receptor that quenches T-cell activation. In order to evaluate whether the LAG3 aptamer was able to increase the threshold of T-cell activation, we performed a proliferation assay on CD4 isolated lymphocytes labeled with CFSE and cultured with splenocytes as feeders and a suboptimal dose of CD3 agonistic antibody. As can be seen in Fig 6A and 6B, CD4 lymphocytes in the presence of LAG3 dimer aptamer (LAG3 Apt1), compared to an equivalent aptamer control (Apt control), showed a higher proliferation rate with a higher amount of cells that underwent up to four divisions. To confirm the activation of T lymphocytes, we performed an in vitro functional assay using isolated CD4 and CD8 lymphocytes stimulated with a suboptimal dose of CD3 agonistic antibodies in the presence of splenocytes as feeders. In this case, T-cell activation was determined by secretion of IFN-γ, quantified by ELISA in the cell supernatant. CD4 and CD8 lymphocytes stimulated with anti-CD3 mAbs in the presence of LAG3 Apt1 exhibit a considerable increased production of IFN-γ as compared with those cells stimulated in the presence of Apt control (with a randomized variable region) or with anti-CD3 alone (Fig 6C and 6D). The results were also confirmed by using specific antigen T lymphocytes, similar to the previous assay in which CD8 lymphocytes isolated from OT-I transgenic mice were activated with SIINFEK as the specific determinant antigens and splenocytes as feeders in the presence of LAG3 Apt1 or Apt control. T-cell activation was determined by INF-γ secretion, measured by ELISA (S4 Fig). The SIINFEKL peptide provides a potent TCR signal as it is a high affinity peptide, but the addition of LAG3-Apt1 increases the production of IFN-γ to a higher extent.

## Discussion

LAG3 is an immune receptor expressed in T lymphocytes, NK cells, Dendritic cells, etc. [20]. This receptor gauges the magnitude of the immune response elicited, in much the same way as it occurs with other immune-checkpoint receptors, e.g. PD1, TIM3, CTLA4, etc. However, contrary to other immune-checkpoints, LAG3 is also a desirable therapeutic target to modulate the immune response either to potentiate it as for instance in cancer immunotherapy, or to ameliorate it as in the case of autoimmune diseases [10–11]. LAG3 antibodies are being tested for these two opposite purposes in clinical trials. In cancer immunotherapy, antibodies that block the receptor on T cells have been developed [6], while depleting antibodies that target activated T cells that express LAG3 are used in clinical trials of autoimmune diseases [11]. Here, we described the first-in-class anti-LAG3 aptamer with potential therapeutic application for cancer immunotherapy or treatment of autoimmune diseases. We have previously shown that aptamers can be easily engineered for different types of applications in a much simpler manner than therapeutic proteins [21] [22] [23]. Aptamer technology is emerging as a novel therapeutic platform in different fields [15], but also in cancer immunotherapy as has been shown over the last few years [24–25]. Aptamers show some differences from antibodies, they are not cell-based products and that is an important advantage as it significantly simplifies the regulatory process facing future clinical trials, thus reducing time and cost of production. There are further advantages (and disadvantages) of aptamers over antibodies, extensively discussed in recent reviews [15].

Herein we described the selection of LAG3 aptamers by using HT-SELEX. The HTS analysis provides a lot of information about the complexity of the library in each round at the time of selection. That data could be used to identify several aptamer clusters and pinpoint conserved sequence motifs in the most populated aptamer clusters. Using this selection approach, we identified three major conserved motifs that could be important for the binding of the aptamer to the target protein LAG3. Surprisingly enough, the aptamer that displays the highest affinity for LAG3 protein contained the three conserved motifs with an apparent Kd of 20 nM determined by P^32^ binding assay and 2.7 nM by SPR. BMS-986016, which is in the antibody used in clinical trials, binds to human LAG-3 with high affinity (Kd = 0.12–0.5 nM) according to WO 2015116539 A1 patent [26]. Looking at the predicted tertiary structure of the aptamer, these motifs were well exposed, indicating that they could be directly involved in the binding to the cognate receptor. Important motifs of just very few nucleotides have been previously identified as determinants for the binding of the aptamer to its target; for instance, the sequence GGUGCU is quite specific of binding to hIgG-Fc [27]. We have also confirmed that, during different SELEX procedures, aptamers that display this motif will bind to IgG Fc tag.

SELEX is a time-consuming, long, tedious process. It involves several rounds of selection against the chosen target, followed by the identification of the most enriched aptamer species. The introduction of deep sequencing allows gathering more data about the selection process. However, that information can sometimes be overwhelming as there might be a large amount of aptamer families. Among all these aptamer families identified by HS-SELEX some of them will be poor binders with low affinities to their targets, some of them will not even bind–sequences that are more prone to be amplified during the (PCR) step or better transcribed–, and sometimes just very few species will be actual high affinity aptamers [16]. The challenge is how to identify those species out of enormous data obtained from HTS. Based on our observations, we elucidated that a possible way to proceed could be to look for conserved motifs detected in low affinity binders and then search for aptamer species that contain as many of those motifs as possible concomitantly, which can then be combined with 3D-structure prediction to corroborate how these conserved motifs are exposed. This work also sheds light on how some aptamers might interact to their targets in a collaborative way, binding simultaneously at different regions of the same protein. Another observation that could support this hypothesis is based on a recent publication from Gilboa’s group. They have described an extremely potent anti-TIM3 blocking aptamer. The biological activity of this new aptamer (TIM3 Apt_b0) surpasses the anti-TIM3 antibody [28]. Looking into the motives of TIM3 Apt_b0 we have found out that it displays three sequence motives that were also present in two different previous aptamers against TIM3 receptor described by our group [29] (S5 Fig).

The variable region of an antibody is also formed by three domains known as complementary determined regions (CDR), and each of these CDR participates in the binding to specific regions of the target. It is too early to assume that some aptamers would interact with their target in a similar way to antibodies–through three binding motives. Further studies of crystallography to determine the real interaction sites will be required to address this question.

## Methods

### HT-SELEX

We performed SELEX procedure as previously described [13]. Selection was performed against the recombinant protein LAG3-IgG, a chimera-recombinant protein that contains the extracellular mouse LAG3 domain fused to human IgG2-Fc (R&D systems, Abingdom, UK). A 31N-nucleotide randomized DNA library flanked with two constant regions was used as a library template GGGAGAGAATTTGGTAATGGGNNNNNNNNNNNNNNNNNNNNNNNNNNNNNNNCCCTTATATCTCTCTCCC; the primers used for library amplification were: Fwd GGGGAATTCTAATACGACTCACTATAGGGAGAGAGATATAAGGG and Rev GGGAGAGAATTTGGTAATGGG. All the transcriptions were performed with 2-Fluoro-UTP and 2-Fluoro-CTP modification in order to improve RNA stability and to confer resistance to RNAses. To that end, we used in vitro transcription with Durascribe kit (Epicentre, Madison, WI, USA). In S1 Table all selection conditions that were performed in each round of SELEX are described. The binding of the library to the target protein was carried out at 37°C in saline buffer (20mM HEPES, 150 mM NaCL, 2mM CaCl2 and 0.01% BSA) and shaken for 30 minutes. mLAG3-Fc chimera recombinant protein was immobilized to protein A sepharose beads (GE Healthcare Bio-science, Uppsala, Sweden). The RNA-bound fraction was extracted via phenol-chloroform-isoamyl alcohol; the recovered RNA was precipitated. Eluted RNA was retro-transcribed and amplified by PCR, as previously described. In each round of SELEX we performed counter-selection against human IgG2 bound to sepharose beads (Sigma Aldrich, Saint Louis, MO).

The DNA-PCR products of rounds 6 and 7 were HT-sequenced by Ion Torrent, and analysis of multiple-sequence alignment was performed by using FASTAptamer software [17] to get the FASTAcount file which indicates the number of reads per million of each selected aptamer and the ranking. With this file we performed a FASTAcluster set at a -d 7 -f 2 to select only the aptamer families that were more abundant. The result of FASTAcluster was again clustered with ClustalW and visualized with Seaview software. All the process was done in a Linux CentOS 6.3 cluster of 4 cores and 64GB. RNA-structure predictions were performed with RNAstructure 5.3 software.

Every selected aptamer was directly generated by transcription from a double-stranded DNA oligonucleotide template which was hybridized from two partially complementary sequences and extended by Klenow reaction. The purified DNA template was transcribed using the T7 Durascribe Kit (Epicentre, Madison, WI) and purified by polyacrylamide gel electrophoresis (PAGE).

### Binding

LAG3Apt was α-ATP P^32^ (PerkinElmer, Madrid, Spain) radiolabeled and used for binding to LAG3 protein (R&D systems, Minneapolis, MN) by nitrocellulose binding assay. The aptamer transcription labeled with α-ATP P^32^ was PAGE purified; we used 100,000 cpm of α-ATP labeled aptamer for the binding assay. The aptamer was denaturalized and refolded by heating at 65°C and cooling to 37°C in buffer containing 150 mmol/l NaCl, 2 mmol/l CaCl2, 20 mmol/l N-2-hydroxyethylpiperazine-N9-2-ethanesufonic acid (HEPES, pH 7.4) and purified in PAGE. The radiolabeled aptamer was incubated with the protein LAG3 at different concentrations in the binding buffer for 30 minutes at 37°C and then added into the dot blot machine including a nitrocellulose filter. The aptamer bound to the protein is quantified in a film. The Kd were determined by using the GraphPad Prims software.

### SPR

Aptamer binding to LAG3 protein was performed by surface plasmon resonance using the ProteOn XPR36 system. The aptamers were biotinylated and immobilized onto the surface of independent channels of a streptavidin sensor chips (Bio-Rad). Different concentration solutions of LAG3-Fc or control Fc protein were injected in running buffer (PBS, 0.005% [v/v] Tween 20, pH 7.4) at a flow rate of 30 μl/min. The signal obtained in the channel immobilized with aptamer was used as reference. The apparent Kd was calculated with GraphPad Prims software from the RU obtained in the equilibrium state at each concentration of recombinant protein LAG3-Fc. Kon and Koff was calculated directly through SPR binding curves using the ProteOn XPR36 software.

### Binding to cells

LAG3 aptamer was labeled by spiking P^32^-αATP in each transcription reaction. To assess the binding of the radioactive aptamer we used OT-I purified T lymphocytes that were pre-activated with 2 μg/ml SIINFEKEL peptide as a stimulus for 72 hours. This treatment induces the expression of LAG3 (confirmed by flow cytometry with an anti-LAG3 antibody). The LAG3-expressing lymphocytes were incubated with the labeled radioactive dimer aptamers, at different cell ratios; as negative control we used the non-activated T lymphocytes which do not express LAG3.

### 3D-Structure prediction with Rosetta

For each modeled aptamer, the most stable 2D structure was generated by using RNAstructure 5.3. [30]. For the generation of 3D RNA structures, the Rosetta package FARFAR [19, 31] was used to build a total of 250 models. Next, the models were sorted by Rosetta energy and selected for 3D structural clustering with an auto-adjusted radius. After examining the cluster population distribution, the lowest Rosetta energy representative model was selected from the most populated cluster for further visual inspection.

### CD4 proliferation assays

CD4 lymphocytes were isolated from spleen, using the Miltenyi negative selection kit (Miltenyi Biotec, Auburn, CA). Purified CD4 lymphocytes were stained with 2 μM of CFSE. CFSE-labeled cells were incubated with irradiated splenocytes (feeder cells) (ratio 10:1) in a 96-well plate coated with anti-CD3 antibody (BD Bioscience, San Jose, CA). Proliferation was determined by flow cytometry on CD4-labeled cells (CD4 antibody, Biolegend, San Diego, CA).

### IFN-ү production assays

CD8 and CD4 lymphocytes were isolated from spleen, using the Miltenyi negative selection kit (Miltenyi Biotec, Auburn, CA). T cells were cultured (10^5^ cells/ml) in complete medium–RPMI-glutamax medium (Invitrogen) supplemented with 10% FCS (Sigma) and 1% penicillin/streptomycin (Invitrogen) in a 96-well plate coated with anti-CD3 agonistic antibody (BD Bioscience, San Jose, CA). T lymphocytes were cultured together with 10^4^ irradiated splenocytes (feeder cells) (ratio 10:1). At day 3, supernatant was harvested and analyzed by ELISA (BD Biosciences) for IFN-ү production.

For antigen-specific TCR stimulation, CD8 lymphocytes isolated from OT-I transgenic mice were cultured (10^5^ cells/ml) in complete medium–RPMI-glutamax medium (Invitrogen) supplemented with 10% FCS (Sigma) and 1% penicillin/streptomycin (Invitrogen) in a 96-well plate coated with 2 μg/ml of SIINFEKL peptide and 10^4^ irradiated splenocytes (feeder cells) (ratio 10:1). Supernatant was collected at day 3 for IFN-γ analysis by ELISA.

Euthanasia of C57BL6 and OT-I transgenic mice was performed by appropriately trained personnel approved on the Animal Protocol using CO2 exposure and follow by cervical dislocation. We obtained the approval for the use of animals by the animal committee CEEA with a protocol number 063/14.

### Statistics

ANOVA test was used as a statistic test in the in vitro assays. All the experiments otherwise mentioned in the text were repeated at least twice.

## Supporting information

S1 Fig Frequencies of each aptamer obtained from the Ion Torrent analysis at R06 (black) and R07 (red) in reads per million (rpm). (JPG)

S2 Fig Predicted secondary structure of LAG3 Apt1 dimer by using RNAstructure software. (JPG)

S3 Fig Expression of LAG3 receptor on naïve and activated lymphocytes measured by flow cytometry. (JPG)

S4 Fig LAG3 Apt1 enhances IFN-γ secretion on TCR-specific stimulated lymphocytes. IFN-γ determined by ELISA in the supernatant of CD8 lymphocytes isolated from OT-I transgenic mice. The lymphocytes were stimulated with SIINFEKL peptide as determinant antigen and cultured with 10:1 ratio of splenocytes as feeders.
(JPG)

S5 Fig Evidence of Conserved Motive Accumulation (CMA) in TIM3 aptamers. TIM3_Apt_b0 described by Gefen et al Mol Ther 2017 which had superior antitumor/blockade effect than the antibody, and TIM3_Apt1 and TIM3_Apt2 described by Hervas et al Oncotarget 2016. The aptamer with higher biological activity TIM3_Apt_b0 display three conserved sequence motives present in TIM3_Apt1 and TIM3_Apt2.
(JPG)

S1 Table LAG3 Aptamers SELEX conditions. (DOC)

S2 Table HTS analysis by FASTAptamer of R06. (XLSX)

S3 Table HTS analysis by FASTAptamer of R07. (XLSX)
