# Supplementary figures and images for: Identification of LAG3 high affinity aptamers by HT-SELEX and Conserved Motif Accumulation (CMA)
Source: PLoS One. 2017 Sep 21;12(9):e0185169. doi: 10.1371/journal.pone.0185169 (PMC5608357; doi:10.1371/journal.pone.0185169)

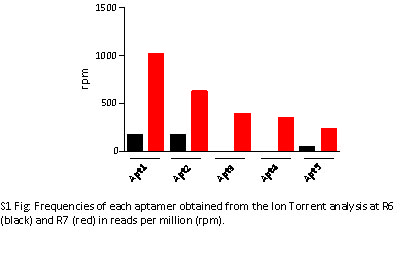

Supplement: S1 Fig — (JPG) [file pone.0185169.s001.jpg]

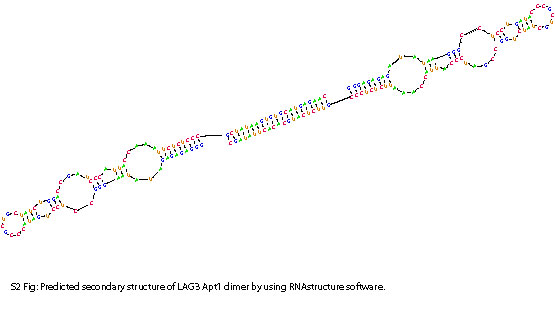

Supplement: S2 Fig — (JPG) [file pone.0185169.s002.jpg]

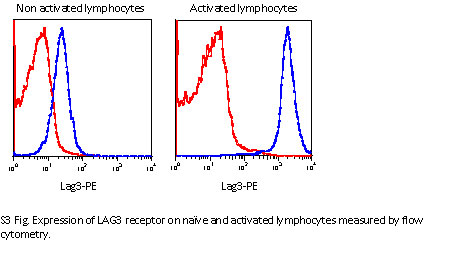

Supplement: S3 Fig — (JPG) [file pone.0185169.s003.jpg]

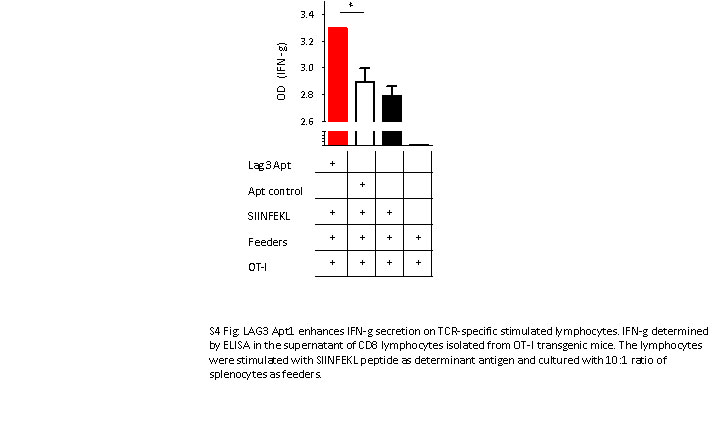

Supplement: S4 Fig — IFN-γ determined by ELISA in the supernatant of CD8 lymphocytes isolated from OT-I transgenic mice. The lymphocytes were stimulated with SIINFEKL peptide as determinant antigen and cultured with 10:1 ratio of splenocytes as feeders. (JPG) [file pone.0185169.s004.jpg]

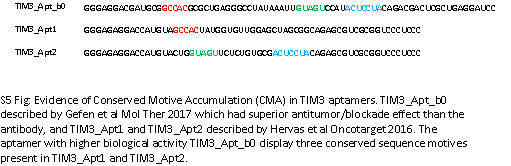

Supplement: S5 Fig — TIM3_Apt_b0 described by Gefen et al Mol Ther 2017 which had superior antitumor/blockade effect than the antibody, and TIM3_Apt1 and TIM3_Apt2 described by Hervas et al Oncotarget 2016. The aptamer with higher biological activity TIM3_Apt_b0 display three conserved sequence motives present in TIM3_Apt1 and TIM3_Apt2. (JPG) [file pone.0185169.s005.jpg]
