# Supplementary material for: Identification of LAG3 high affinity aptamers by HT-SELEX and Conserved Motif Accumulation (CMA)
Source: PLoS One. 2017 Sep 21;12(9):e0185169. doi: 10.1371/journal.pone.0185169 (PMC5608357; doi:10.1371/journal.pone.0185169)
Supplement: S1 Table — (DOC) [file pone.0185169.s006.doc]

| Round | **RNA (µM)** | **LAG3-Fc (nM)** | **IgG**  **(nM)** | **Time wash** |
| --- | --- | --- | --- | --- |
| 1 | 2 | 1 | 1 | 2 washes 30s |
| 2 | 1 | 1 | 1 | 3 washes 1 min |
| 3 | 0.5 | 0.8 | 1 | 3 washes 1 min |
| 4 | 0.25 | 0.4 | 1 | 3 washes 2 min |
| 5 | 0.125 | 0.4 | 1 | 3 washes 3 min |
| 6 | 0.125 | 0.2 | 1 | 3 washes 3 min |
| 7 | 0.05 | 0.2 | 1 | 3 washes 5 min |

S1 Table: LAG3 Aptamers SELEX conditions
